# To know where we came from

**DOI:** 10.1002/pne2.12032

**Published:** 2020-08-08

**Authors:** Randi Dovland Andersen

**Affiliations:** ^1^ Department of Research Telemark Hospital Skien Norway

In this first thematic issue of Paediatric and Neonatal Pain, four invited articles together paint a picture of the emergence and development of the pediatric pain research field, with an emphasis on infant pain, from different personal, professional, and methodological perspectives.

Planning this issue, there were several influential researchers to choose from, but we invited Kanwaljeet “Sunny” Anand as a pediatric anesthesiologist to represent the medical perspective, C. Céleste Johnston nursing, Kenneth Craig pediatric psychology, and Ruth Grunau neuroscience.

Pain in the youngest children underwent a paradigm shift in the 1980s. In their seminal paper from 1987, Anand and colleagues demonstrated the consequences of surgery without anesthesia on infant morbidity and mortality.^1^ Still, as important as research findings in instigating changes, was personal stories like the one of prematurely born Jeffery Lawson, brought forward by his mother Jill when Jeffery died 5 months after undergoing open‐heart surgery without anesthesia. Jill Lawson brought awareness to the lack of pain management for infants and helped instigate the necessary changes in clinical practice. This illustrates the importance of groundbreaking research to challenge current knowledge, but also the powerful effect of a personal story. While research findings mainly trigger our intellect and cognitive understanding, stories speak directly to our emotions and often act as a powerful catalyst for change. The articles in this issue weave together both perspectives.

In their paper, Anand and coauthors identified landmark publications on infant pain management through a bibliometric analysis of their relative importance, measured as frequency of citations in the years after publication.^2^ Not surprisingly, all invited authors were on the top‐20 list with Anand and colleagues seminal 1987 paper as number one of the list.

Céleste Johnston describes how her research program unfolded from a clinical nursing question on how to measure pain in infants and into researching various aspects of assessment and management of pain. In recent years, her research has addressed the involvement of mothers in alleviation of pain,^3^ illustrating the growing awareness that to understand and be able to alleviate pain we have to take into consideration the social context.

It is impossible to talk about the social context of pain without referring to Kenneth Craig and his foundational work on pain as a social experience. In his paper, Craig describes his efforts over several decades to understand the social dimension of pain^4^ as it is conceptualized in the social communication model of pain.^5^

Ruth Grunau describes how she started her career as a PhD student with Kenneth Craig, using facial action coding to study facial expression of pain empirically. She moved on to establish a multidisciplinary research program on the long‐term effects of repetitive pain exposure in infants born very preterm.^6^

The four of them have published groundbreaking research that has helped move the field forward, but the overall impact of their life's work may be more easily comprehended when put into context and/or told as a story. However, their stories are about more than the research findings in themselves. A common theme that emerges is that of collaboration, connections, and building a community. They have worked together on different research projects, for example Anand and Craig's work on expending the definition of pain to include also those unable to express their pain verbally.^7^ Each of them has helped bring forward the next generations of pediatric pain scientists and equip them for their further work in the field. Several former trainees, now world‐renowned experts themselves, are mentioned in the articles. Johnston, Craig, and Grunau were part of a transdisciplinary team lead by Dr Patrick McGrath that established and grew the Canadian‐based research‐training consortium Pain in Child Health (PICH). For the past 18 years and counting, PICH has developed a community of Canadian and international highly qualified pediatric pain researchers.^8^ Finally, all seem to agree that the future of pediatric pain research is hopeful and safe in the hands of the current generation and the researchers they helped train. I hope that some of that new research finds its way onto the pages of this journal. Paediatric and Neonatal Pain was recently established, and it is the only journal focusing exclusively on pain in children. We rely on the pediatric pain community to help grow it as a go‐to source and outlet for our work.

Together, the articles in this special issue provide both experienced and new researchers with a unique overview of the development of the pediatric pain research field, seen through the eyes and illustrated by the work of four of the pioneers in the field. Thank you for leading the way and sharing your perspectives. I truly believe that a keen understanding of what came before us and how we came to be where we are today is a necessary foundation for current research and for shaping the directions for future developments of the pediatric pain field. To quote the late American civil rights leader Joseph Lowery:

If you don't know where you come from, it's difficult to determine where you are. It's even more difficult to plan where you are going
